# The Yeast Transcription Factor Crz1 Is Activated by Light in a Ca^2+^/Calcineurin-Dependent and PKA-Independent Manner

**DOI:** 10.1371/journal.pone.0053404

**Published:** 2013-01-15

**Authors:** Kristofer Bodvard, Anna Jörhov, Anders Blomberg, Mikael Molin, Mikael Käll

**Affiliations:** 1 Department of Applied Physics, Chalmers University of Technology, Göteborg, Sweden; 2 Department of Chemistry and Molecular Biology, University of Gothenburg, Göteborg, Sweden; Université de Nice-CNRS, France

## Abstract

Light in the visible range can be stressful to non-photosynthetic organisms. The yeast *Saccharomyces cerevisiae* has earlier been reported to respond to blue light via activation of the stress-regulated transcription factor Msn2p. Environmental changes also induce activation of calcineurin, a Ca^2+^/calmodulin dependent phosphatase, which in turn controls gene transcription by dephosphorylating the transcription factor Crz1p. We investigated the connection between cellular stress caused by blue light and Ca^2+^ signalling in yeast by monitoring the nuclear localization dynamics of Crz1p, Msn2p and Msn4p. The three proteins exhibit distinctly different stress responses in relation to light exposure. Msn2p, and to a lesser degree Msn4p, oscillate rapidly between the nucleus and the cytoplasm in an apparently stochastic fashion. Crz1p, in contrast, displays a rapid and permanent nuclear localization induced by illumination, which triggers Crz1p-dependent transcription of its target gene *CMK2*. Moreover, increased extracellular Ca^2+^ levels stimulates the light-induced responses of all three transcription factors, e.g. Crz1p localizes much quicker to the nucleus and a larger fraction of cells exhibits permanent Msn2p nuclear localization at higher Ca^2+^ concentration. Studies in mutants lacking Ca^2+^ transporters indicate that influx of extracellular Ca^2+^ is crucial for the initial stages of light-induced Crz1p nuclear localization, while mobilization of intracellular Ca^2+^ stores appears necessary for a sustained response. Importantly, we found that Crz1p nuclear localization is dependent on calcineurin and the carrier protein Nmd5p, while not being affected by increased protein kinase A activity (PKA), which strongly inhibits light-induced nuclear localization of Msn2/4p. We conclude that the two central signalling pathways, cAMP-PKA-Msn2/4 and Ca^2+^-calcineurin-Crz1, are both activated by blue light illumination.

## Introduction

Yeast cells are continuously exposed to a variety of external conditions that they need to cope with to maximize growth and survival in their natural environment. To maintain optimal internal conditions (i.e. homeostasis) a diverse set of mechanisms, control functions and strategies have evolved. In contrast to higher eukaryotes, unicellular organisms like the budding yeast *Saccharomyces cerevisiae* cannot utilize specialized tissue for protection against a wide range of environmental challenges, such as variation in temperature, pH, toxic agents, osmolarity, and UV or visible light illumination. Instead, the whole organism must be able to quickly adapt to environmental changes to counter damage. Naturally, one set of mechanisms optimized to counteract a certain external stress might not be appropriate for a different type of disturbance, i.e. many of the stress responses are specific and often highly complex. For example, signalling pathways react differently to different types of stresses and their responses may also depend on previous cellular “states” [1]. In general, the structure of the signalling pathways that control stress-induced gene expression in yeast are rather well characterized, but much less is known about the dynamic behaviour of the signalling components, in particular at the single cell level. To be able to understand the molecular mechanisms by which yeast cells regulate stress responses, it is important to determine the spatial and temporal dynamics of the transcription factors that are involved in initiating and controlling stress-induced gene expression. Previous studies have revealed that both the zinc finger transcription factor Crz1p [2] and the general stress response transcription factors Msn2p and Msn4p control gene expression through nucleocytoplasmic oscillations [2], [3], [4], [5], [6], [7], [8]. The three transcription factors have also been implicated in the response to multiple environmental challenges [1], [9], [10], [11], [12]. In this contribution, we focus on the connection between Ca^2+^-signalling and visible light illumination and report on a comparative study of the temporal localization dynamics of these transcription factors in individual cells subject to the same level of light-induced stress.

Stress responses in yeast involve multiple integrated signalling cascades [13] that can be broadly divided into a general component, the environmental stress response (ESR) [10], [11], [14], and other systems that respond to more specific environmental changes [11], [12], [15]. One of the most universal signalling molecules is Ca^2+^, which fulfils critical functions in both uni- and multi-cellular organisms [16]. Ca^2+^-dependent signalling pathways can either be induced by increased cytosolic levels of Ca^2+^ recruited from secretory compartments, which in eukaryotic cells contain high concentrations of Ca^2+^, or from increased influx from extracellular sources [17]. In *S. cerevisiae* Ca^2+^ signalling is critical for cell survival during exposure to a variety of environmental stresses, including high salt concentrations, alkaline pH, and cell wall damage. These conditions cause an increase in cytosolic Ca^2+^ [18]. Under normal conditions the cytosolic Ca^2+^ concentration is held within 50–200 nM in actively budding cells, even when the extracellular conditions vary from <1 µM to >100 mM [19]. The intracellular concentration is controlled by a well-characterized homeostatic system consisting of Ca^2+^ pumps, exchangers and channels [18], [20]. When the cytosolic Ca^2+^ levels increase, the Ca^2+^ sensor protein calmodulin binds to and activates calcineurin, a well-conserved protein phosphatase. Calcineurin, in turn, dephosphorylates Crz1p [21], which then translocates from the cytosol to the nucleus, where it binds to promoter sequences called CDRE (calcineurin-dependent response element) and activates roughly 160 genes involved in the adaptation to stress [12], [22]. These genes are involved in a number of different functions, including cell wall integrity and ion homeostasis [18], as well as genes induced during exposure to e.g. H_2_O_2_ and heat shock [12].

The transcription factors Msn2p and Msn4p [23] have been identified as the main general inducers of the response to environmental stresses in budding yeast [13]. During stress, Msn2p and Msn4p shuttle to the nucleus where they affect the expression of roughly 180 genes [10], [11] by binding to STREs (stress response elements) in target gene promoters. Several stresses are known to induce nucleocytoplasmic oscillations of Msn2p, including high concentrations of extracellular Ca^2+^ [2], calorie restriction (low glucose levels 0.1%) [5], [8], oxidative and osmotic stress [5], as well as light [3], [4], [6], [7]. The localization to the nucleus is negatively correlated to both cAMP levels and protein kinase A (PKA) activity [24], [25]. Msn2p and Msn4p exhibit 41% identity at the amino acid level and are similar in size [23]. They were initially thought to fulfil similar functions in the stress response [23], [26], but more recent studies instead indicate that Msn2p and Msn4p affect gene induction differently [27], [28], in a both gene- and stress-condition specific manner [1]. Moreover, Msn2p and Msn4p target genes do not seem to be essential for survival against a single severe stress. Instead, they seem to be more important for survival to a secondary stress after a mild non-lethal primary stress, so called acquired stress tolerance [1].

By following the temporal and spatial dynamics of the transcription factor Msn2p tagged with green fluorescent protein (GFP) using fluorescence microscopy, we previously demonstrated that continuous exposure to blue light leads to cumulative cellular stress, most likely due to accumulation of toxic photoproducts [3]. The light-induced stress leads to seemingly stochastic displacement of the Msn2p population in a cell from the cytoplasm to the nucleus that depends on several factors, including light intensity [3], [4], [6], [7]. Here we report that also Crz1p is activated during illumination, i.e. it localizes to the nucleus and activates gene expression. We quantitatively characterize its temporal localization pattern for a cell population (N≈100) and compare the results to the nucleocytoplasmic shuttling observed for Msn2p and Msn4p under identical illumination conditions. Although the three transcription factors respond at the same level of light intensity, they behave distinctly differently in the sense that Crz1p exhibits an essentially permanent localization to the nucleus whereas both Msn2p and Msn4p oscillate. The behaviour observed for Crz1p is also drastically different to the previously reported response following exposure to high extracellular Ca^2+^ levels [2], which leads to stochastic “bursts” of nuclear localization. In addition, we report that light-induced Crz1p nuclear localization is dependent on calcineurin but independent of PKA activity. Moreover, the nuclear localization responses of Crz1p, and to some degree Msn2p, were found to be strongly stimulated by extracellular Ca^2+^. Apart from delineating a novel role for Crz1p in the cellular response to illumination, our results highlight the light-induced activation of two central signalling pathways, cAMP-PKA-Msn2/4 and Ca^2+^-calcineurin-Crz1.

## Materials and Methods

### 2.1 Cells and Cultivation

Haploid *S. cerevisiae* (BY4742) yeast cells with the genotype *MATα his3Δ1 leu2Δ0 lys2Δ0 ura3Δ0* were used throughout this study. The single deletion strains, except for *nmd5*Δ (see below for details), were acquired from the EUROSCARF yeast deletion collection [29]. Haploid wild-type and single deletion strains were transformed with plasmids expressing Msn2-GFP, Msn4-GFP or Crz1-GFP. Msn2-GFP was expressed from the plasmid pAMG [25] based on YCplac111, controlled by the *ADH1* promoter and containing a *LEU2* marker. Crz1-GFP was expressed from the pKK249 plasmid [30], controlled by the *MET25* promoter and containing a *URA3* marker. Msn4-GFP was expressed in the same way as Msn2-GFP using the plasmid pGR247 [6], which was derived from pAMG in which the *MSN2* ORF had been replaced by the *MSN4* ORF from the plasmid pAL32-45. Strain YAJ92 (*nmd5Δ*) was created by deleting *NMD5* in strain BY4742 by the one-step PCR targeted gene disruption method [31] using the following primer oligonucleotides (*nmd5Δ* fw TTTCAGCATAATCAATCATTTTTCGTCTGGATATTTGACACAATTTTGATTTTGACGAAGACATTTTATTCTCGATAATACGATTTAGGTGACACTATAG, *nmd5Δ* rev TTGAGCATAATATCCTCTCTCTTCTATCTAAATTATGTAATCCAGTGTTTGTTTCAATATACAATCGCCATTTAATTCAAATACGACTCACTATAGGGAG) and plasmid pFA6-kanMX4 as a template. Correct integration was verified by diagnostic PCR. Strains YAJ85 (wild-type Cmk2-GFP) and YAJ89 (*crz1Δ* Cmk2-GFP) were generated by crossing BY4741 Cmk2-GFP to BY4742 *crz1Δ::kanMX4* (both from EUROSCARF) and selecting for methionine prototrophic, histidine prototrophic, lysine auxotrophic and G418 sensitive (YAJ85) or G418 resistant (YAJ89) progeny. YAJ85 is *MAT*a and YAJ89 is *MATα*.

Strains were grown at 30°C in synthetic defined medium containing Yeast Nitrogen Base (Formedium™) with ammonium sulphate (0.5%), glucose (2%) and complete supplement mixture without the appropriate amino acids and nucleotide bases (-leucine for Msn2-GFP and Msn4-GFP experiments, and -methionine and -uracil for Crz1-GFP experiments), buffered to pH 5.8 with 1% succinic acid and 0.6% NaOH. The same medium was used in experiments with different [Ca^2+^], but ammonium sulphate was replaced with 3.5 g/l ammonium chloride (Sigma) and CaCl_2_ (Sigma) was used to set the [Ca^2+^]. Medium without CaCl_2_ still contained trace amounts of Ca^2+^ originating from calcium pantothenate (0.4 mg/l ∼ 0.8×10^−3^ mM). Overnight cultures were inoculated in the morning and grown to an OD_600_ of 0.4–0.5 at the start of the microscopy experiment. Where indicated addition of EGTA (ethylene glycol-bis(2-aminoethylether)-N,N,N’,N’-tetraacetic acid) to a final concentration of 5 mM was made from a stock solution of 200 mM (pH 8.0).

### 2.2 Fluorescence Microscopy

Images were acquired using an automated epi-fluorescence microscope (TE2000E-PFS, Nikon Instruments) equipped with a 60X oil immersion objective (NA1.4, Plan Apochromatic, Nikon Instruments) and an electron-multiplying CCD camera (iXon DU-885-CS0-#VP, Andor Technology). GFP excitation and simultaneous stress induction were performed by continuous illumination with blue light (450–490 nm) as previously described [3]. Brightfield images, with a small focus offset, were collected once every minute to facilitate cell recognition during image analysis. The light intensities were set to 26 µW (0.10 J·cm^−2^·s^−1^), 58 µW (0.23 J·cm^−2^·s^−1^) and 115 µW (0.45 J·cm^−2^·s^−1^) by combining different neutral density filters and held constant throughout the experiments. The illuminated area was 2.57×10^−4^ cm^2^. Fluorescence images were captured continuously for at least 60 minutes in one focal plane with an acquisition time of 800 ms - 4 s, using the software NIS-elements. The temporal resolution corresponded to roughly 4 s for all transcription factor experiments. During microscopy experiments the cells were kept in a FCS2 perfusion chamber (Bioptechs Inc.) at 28°C to avoid any heat-induced stress response. Fresh medium was added to the chamber just before the start of the experiment. The cover glasses had been pre-coated for 1.5 hours with the lectin protein Concanavalin A (ConA), 0.5 µg/µl in 0.01 M PBS, in order to immobilize yeast cells on the surface. The coating did not induce any stress response. After each experiment, cells surrounding the illuminated area were imaged in order to ensure that only cells that had been illuminated exhibited nuclear localization, thus excluding possible errors in the setup or uncontrollable factors that could lead to stress induction. Each condition and strain was run at least twice and results are based on 80–258 cells, depending on the number of cells captured during each run.

In the case of Cmk2-GFP, cells were illuminated during 40 minutes (115 µW). To track fluorescence changes after light-induced stress images were captured every 10 minutes for 5 hours, leaving the cells in the dark in between images. Where indicated the transcription inhibitor 1,10-phenanthroline was added to the medium just prior to illumination to a final concentration of 100 µg/ml from a stock solution of 100 mg/ml (in ethanol).

### 2.3 Image Analysis

Images were analysed using the software CellStat [32], which identifies cell contours and extracts fluorescent data based on the algorithms described in [33]. In a Gaussian smoothed version of the original fluorescent image, the brightest pixel and its surrounding pixels within a 3-pixel radius was defined as the nucleus. To reduce fluctuations due to e.g. changes in vacuole size or slightly erroneous cell contours, the 90 brightest pixels from the remaining cell pixels were considered to represent the cytosol. To evaluate transcription factor nucleocytoplasmic localization dynamics, the ratio for each time point between the median intensity in the nucleus and the median intensity in the cytosol were used. The localization trajectory used in signal analysis was defined by subtracting one from this ratio, so that no nuclear localization was set to approximately zero. The nuclear localization trajectories can be found in the supplementary data sheets.

To evaluate the impact of higher than native Crz1p levels on the light-induced response we compared the responses of cells grown with or without methionine (expression of Crz1-GFP is controlled by the *MET25* promoter in pKK249). In medium containing methionine the GFP fluorescence was at least 4-fold lower than in the medium used for the present Crz1p studies lacking methionine, but we could not see any difference in the Crz1p nuclear localization pattern upon illumination (data not shown).

To evaluate Cmk2-GFP changes, the mean fluorescence in each cell (Cell) was used. The mean background (BG) value was calculated from an arbitrary area without cells. The total population average response was then calculated 
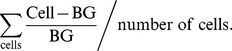


### 2.4 Signal Analysis

Nuclear localizations were identified by thresholding the localization trajectory. A moving average filter of 5 frames first filtered the localization trajectory and then a binary signal was constructed by applying a threshold of 0.28. Values above the threshold represented nuclear localization. Periods of nuclear localization and no nuclear localization of less than 5 frames (∼20 s) were turned into no nuclear localization and nuclear localization, respectively.

Throughout the paper, the Mann–Whitney U-test [34] has been used to assess whether a data set takes larger values than another data set. The test is based on ranks and is therefore non-parametric, i.e. makes no assumptions of the data belonging to a particular distribution, which makes it an appropriate choice in this application where in particular a Gaussian distribution cannot be assumed.

## Results

### 3.1 Exposure to Continuous Blue Light Induces Crz1p Nuclear Localization with Distinctly Different Nucleocytoplasmic Localization Pattern Compared to Msn2p and Msn4p

**Figure 1:**
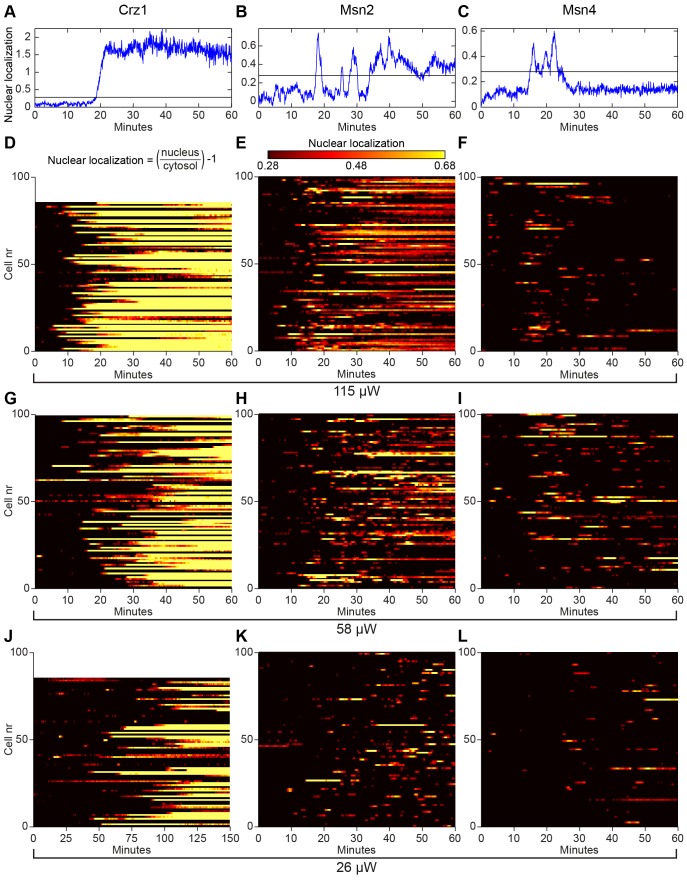
Nucleocytoplasmic localization responses of Crz1p, Msn2p and Msn4p during continuous exposure to blue light. (A)–(C) Nuclear localization trajectories in a single cell and (D)–(L) temporal nucleocytoplasmic localization profiles for populations of 80–100 cells extracted from fluorescence microscopy images for Crz1-GFP (left column), Msn2-GFP (middle column) and Msn4-GFP (right column) at three different light intensities (rows). (A)–(C) The horizontal line represents the threshold used for determining nuclear localization. (D)–(L) Nuclear localization (colour scale) visualizes the relative fluorescence intensity between the nucleus and the cytoplasm in individual cells ((I_nucleus_/I_cytosol_)−1). Crz1p permanently entered the nucleus in a comparatively uniform fashion throughout the whole cell population and exhibited occasionally only one or two oscillations in some cells prior to permanent nuclear localization (A, D, G, and J). Note the different time scale in (J). Msn2p and Msn4p exhibited nucleocytoplasmic oscillations, where the oscillatory response of Msn2p was much more pronounced. A subpopulation of cells showed permanent Msn2p nuclear localization after a period of oscillations (mainly seen in B and E).

In this study we found that the yeast transcription factor Crz1p, a target for the Ca^2+^/calmodulin dependent phosphatase calcineurin, localized rapidly to the nucleus in almost the whole cell population during exposure to continuous blue light (450–490 nm). Figs. 1A–C show a typical localization trajectory (degree of nuclear localization in an individual cell vs. time) for Crz1p, Msn2p and Msn4p at the highest tested light intensity (115 µW). Crz1p migrates to the nucleus after about 20 minutes of illumination and then stays there for the remaining duration of the experiment (Fig. 1A). In contrast, Msn2p oscillates between the cytoplasm and the nucleus in an apparently stochastic manner, as previously reported [4], [6], before also localizing permanently to the nucleus (Fig. 1B). The three successive localization states observed for Msn2p, that is, cytoplasmic localization, nucleocytoplasmic oscillation and permanent nuclear localization, is in good agreement with our previous report on this phenomenon [3]. The localization trajectory for Msn4p (Fig. 1C) is quite different from both Msn2p and Crz1p in the sense that it exhibits an oscillatory response, but never displays sustained nuclear localization.

**Figure 2:**
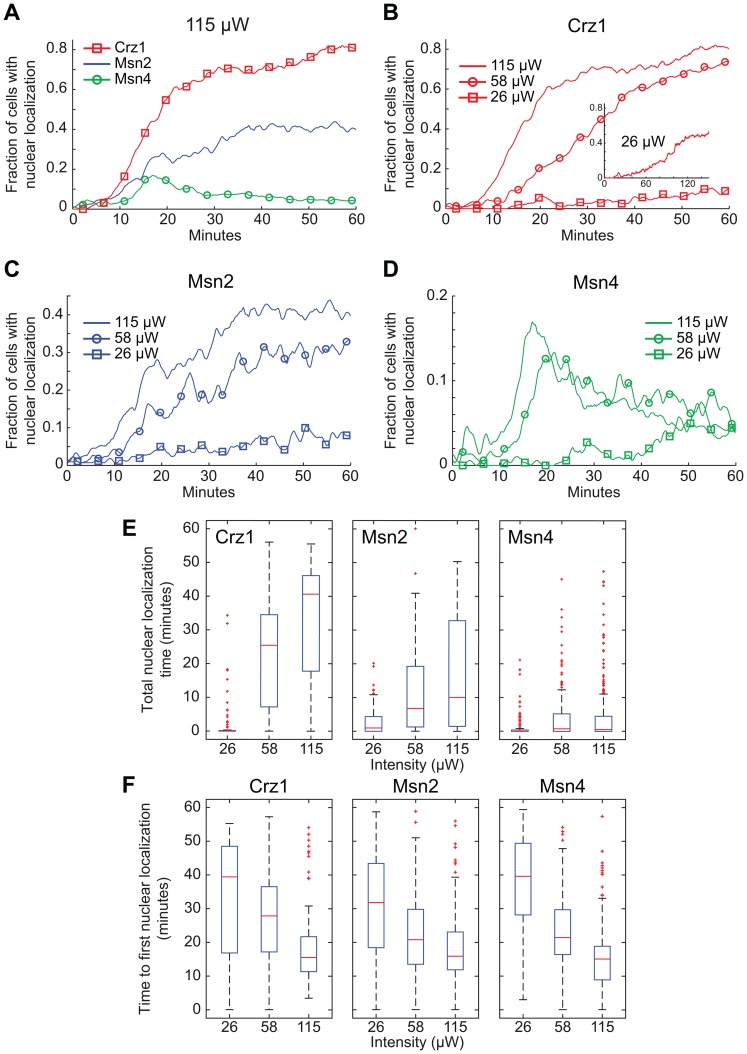
Quantitative analyses of the effects of illumination on nuclear localization of Crz1p, Msn2p, and Msn4p. (A)–(D) Fraction of cells with transcription factors localized to the nucleus at different light intensities. Inset (B): Crz1-GFP exposed to 26 µW during 150 minutes. (E) The total time in the nucleus for the transcription factors Crz1p, Msn2p and Msn4p, during 60 minutes of continuous light exposure at different intensities. There is no statistically significant difference between the responses at the two highest light intensities for Msn4p (p = 0.2145). (F) Time to first nuclear localization of Crz1p, Msn2p and Msn4p at different light intensities. The changes in time to the first nuclear localization as a function of light intensity are statistically significant (Msn2p 58 µW vs. 115 µW p = 0.011, others p<0.001), except for Crz1p 26 µW vs. 58 µW (p = 0.109). Note that Crz1p has only entered the nucleus in ∼25% of the cells at 26 µW during the first 60 minutes considered here, giving rise to a relatively fast median value of 39 minutes. Statistical tests were carried out using the Mann–Whitney U-test.

As detailed in paragraph 2.4, we quantified the degree of transcription factor nuclear localization in each individual cell by thresholding the localization trajectory (examples of thresholds shown in Figs. 1A–C). This allows us to illustrate the population behaviour through colour plots (Figs. 1D–L) and to analyse the degree of nuclear localization quantitatively (Fig. 2). The overall degree of nuclear localization decreased with decreasing light intensity for all three transcription factors, as expected (Figs. 1D-L). This effect is quantified in Figs. 2A–D, which show that the fraction of cells with nuclear localization decreased with decreasing light intensity for any a given time point. However, there are also clear differences on the overall shape of the curves between the three transcription factors. At the highest light intensity, 115 µW, all transcription factors responded after roughly 10 minutes (Fig. 2A). Both Crz1p and Msn2p reached a plateau after 20–30 minutes of exposure, albeit at completely different levels, ∼75% and ∼40%, respectively. Msn4p, on the other hand, exhibited a transient behaviour with a peak of ∼17% after 17 minutes, after which only a few percent of the cells displayed nuclear localization (seen also in Figs. 1F and I). At the intermediate light intensity, 58 µW, peak Msn4p nuclear localization was shifted towards a later time-point (Fig. 2D).

Figs. 2E–F show two additional parameters that can be used to quantify the nuclear localization trajectories; the total time (out of 60 minutes) that each transcription factors spends in the nucleus and the time from the start of the light exposure until the first nuclear localization is detected. Crz1p and Msn2p obviously respond in proportion to light intensity in both cases, i.e. they spent longer time in the nucleus as the light intensity increased (Fig. 2E) and the lag time before the first nuclear localization event was detected decreased with higher light intensity (Fig. 2F). The latter effect was also observed for Msn4p, but no statistical difference in total nuclear localization time could be observed between 58 µW and 115 µW (Fig. 2E).

Both Msn2p and Crz1p spent considerably longer time in the nucleus compared to Msn4p (Fig. 2E). The low median value for Msn4p is partly due to the fact that only ∼50% of the cells responded at all during this time period, even though a typical single Msn4p oscillation has a duration of 2–3 minutes (Fig. 1C). Crz1p and Msn2p, on the other hand, exhibited nuclear localization in >80% of the cells (115 µW), although the percentage of cells with Crz1p nuclear localization was drastically reduced for the lowest intensity. However, when cells were instead followed for 150 minutes, Crz1p exhibited nuclear localization in >55% of the cells (inset Fig. 2B). The median time to first nuclear localization then increases to 79 minutes. Such a distribution is significantly different compared to higher light intensities (p<10^−11^, Mann-Whitney U-test). Moreover, note that the nuclear response to illumination became more homogenous on a population level at higher light intensities, indicated by the steeper slope in e.g. Fig. 2B.

### 3.2 The Light-induced Response of Crz1p is Calcineurin Dependent and Independent of PKA Activity

**Figure 3:**
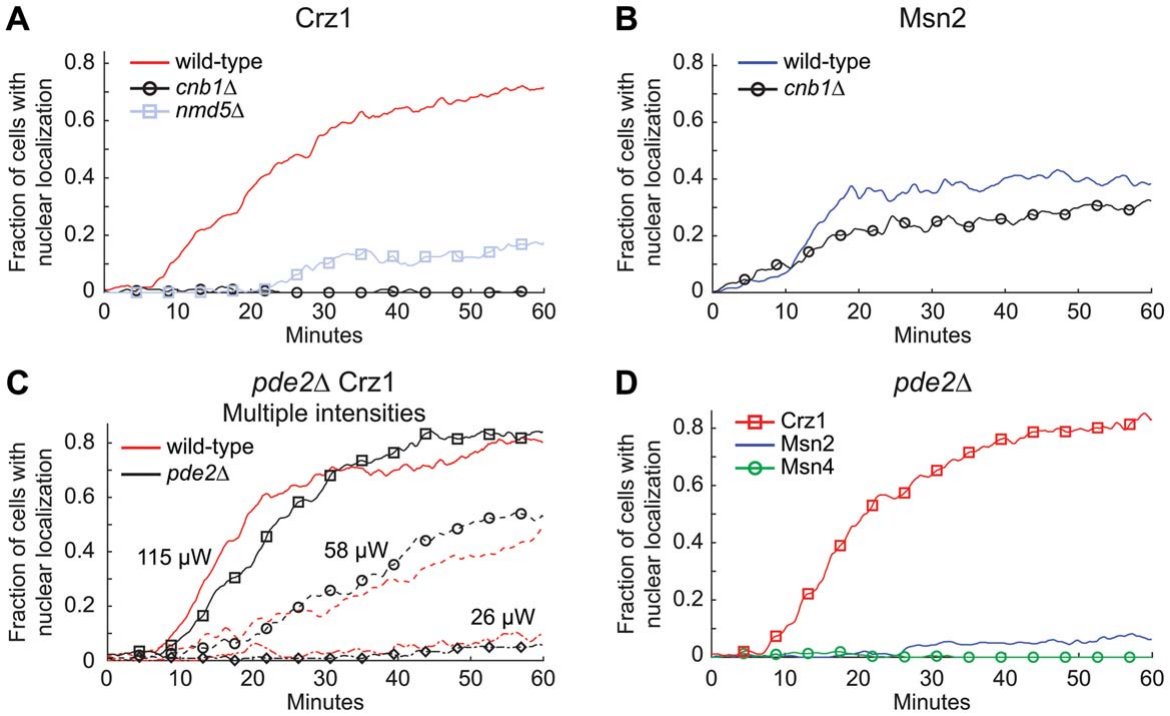
Transcription factor nuclear localization dependence on calcineurin and protein kinase A activity. (A) Fraction of cells with Crz1-GFP localized to the nucleus. In both *cnb1Δ* and *nmd5Δ* strains Crz1p nuclear localization was drastically reduced. (B) Fraction of cells with Msn2-GFP localized to the nucleus in a *cnb1Δ* strain. (C) Fraction of cells with Crz1-GFP nuclear localization in a *pde2Δ* strain at different light intensities (115 µW, 58 µW and 26 µW). (D) Fraction of cells with transcription factor nuclear localization in *pde2Δ* mutants (115 µW). Corresponding wild-type/reference experiments are shown for each sample set.

Several studies have revealed that Crz1p nuclear localization is dependent on the cytosolic levels of Ca^2+^ [18], [20], [21]. Our findings of an essentially permanent nuclear localization of Crz1p during illumination are in contrast to an earlier report of stochastic “bursts” of Crz1p nuclear localization following Ca^2+^ stress (50–300 mM) [2]. This raises interesting questions regarding Ca^2+^-dependent signalling during illumination and we wanted to further investigate the role played by different components. Calcineurin is a calmodulin dependent phosphatase that controls the localization of Crz1p under several stress conditions [21], [35] and we therefore examined the light-induced response of Crz1-GFP in a mutant strain lacking the regulatory domain of calcineurin, *CNB1* [36], [37]. The light-induced Crz1p response was clearly dependent on this phosphatase since in the *cnb1Δ* strain no cells displayed nuclear localization of Crz1p even after 60 minutes at the highest light intensity (115 µW; Fig. 3A). The same experiment was repeated for Msn2-GFP (Fig. 3B). The results in the *cnb1Δ* strain indicated no significant decrease in Msn2-GFP total nuclear localization time (p = 0.08, Mann-Whitney U-test (Fig. S1)).

Previous studies have shown that nuclear accumulation of Crz1p in response to high extracellular Ca^2+^ requires the nuclear importin Nmd5p [38]. We evaluated if Nmd5p was important also for nuclear localization during illumination and found that in an *nmd5Δ* strain the initial Crz1p nuclear localization was almost lost (Fig. 3A), although a few cells appeared to respond after ∼25–30 minutes or longer.

Under certain conditions Crz1p is also negatively regulated by the casein kinase I homolog Hrr25p [39] and PKA [30]. PKA directly phosphorylates the NLS of Crz1p and in that way counteracts calcineurin activity. Thereby, Crz1p integrates stress-induced signalling via both these two signalling routes. Since Crz1p, Msn2p and Msn4p were all affected by illumination, a possible mechanistic hypothesis for cross-coordination is that blue light exposure lowers PKA activity. This in turn would alter the balance between the activity of PKA and calcineurin and allows for increased Crz1p nuclear localization. To test this hypothesis, the GFP tagged transcription factors were expressed in a *pde2Δ* strain. Pde2p is the cAMP phosphodiesterase with the highest affinity for cAMP, which converts cAMP to AMP [40], [41]. Deletion of *PDE2* yields a strain with high cAMP levels and thus high PKA activity [42], which we previously showed drastically diminishes Msn2p nuclear localization during illumination [3]. However, Crz1p still entered the nucleus in the *pde2Δ* strain as in the wild-type strain at all light intensities tested (Fig. 3C). As with Msn2p, Msn4p nuclear localization was inhibited in the *pde2Δ* strain during illumination. The number of cells that responded to illumination at all (from a total of >150 cells in two experiments) decreased from 50% to 5% and from >80% to 20% in the cases of Msn4p and Msn2p, respectively. In addition, the few mutant cells that did respond showed in general much weaker responses (Fig. 3D and Fig. S2) compared to wild-type cells (Figs. 1 and 2A). Altogether these data show that light-induced Crz1p nuclear localization is dependent on calcineurin activity and independent of PKA activity. In addition, we conclude that at least two signalling pathways are involved in activating transcription factors in the response to light-induced stress.

### 3.3 Increased Extracellular [Ca^2+^] Strongly Stimulates Crz1p but Only Moderately Increases Nuclear Localization of Msn2p and Msn4p

**Figure 4:**
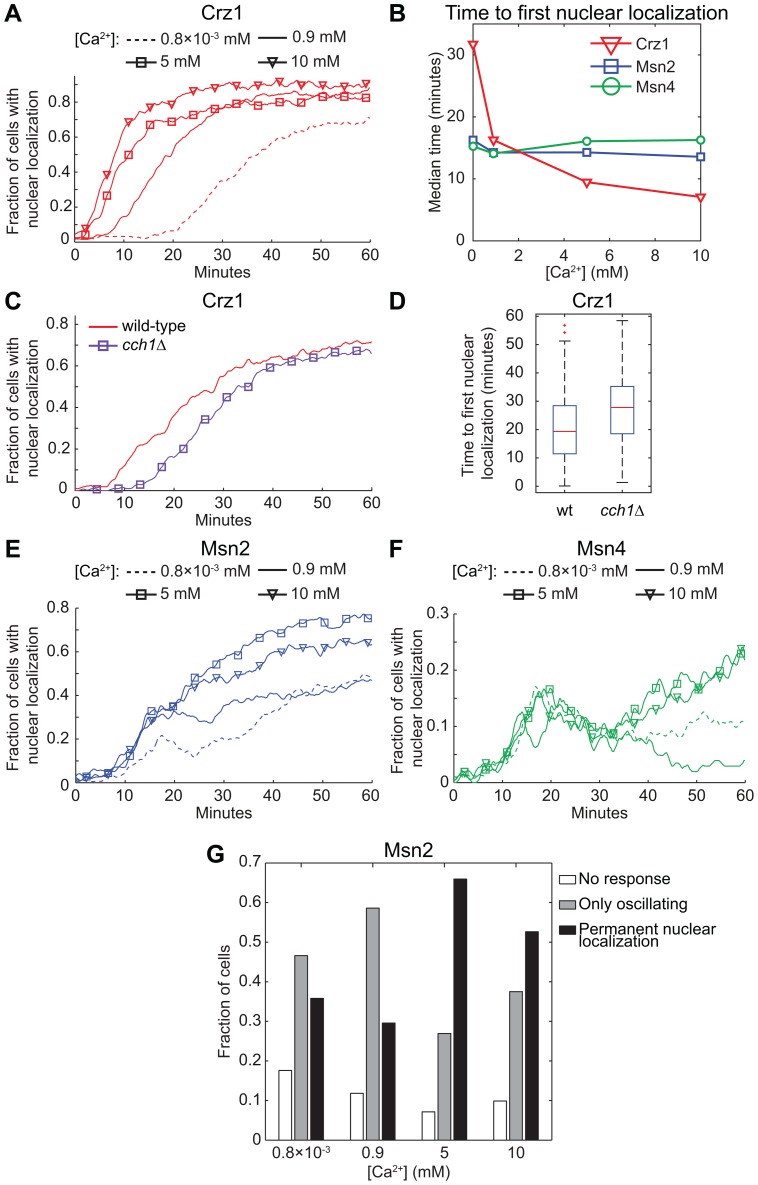
Effect of the extracellular [Ca^2+^] and cellular influx on Crz1p, Msn2p and Msn4p localization in response to light. Fraction of cells with the transcription factors (A) Crz1-GFP, (E) Msn2-GFP and (F) Msn4-GFP localized to the nucleus as a function of time during continuous exposure to blue light (450–490 nm, 115 µW). (C)–(D) A delayed nuclear localization response was found in cells lacking the plasma membrane calcium channel *CCH1*. The difference is statistically significant (p<0.0001). (A)–(B) and (E)–(F) Cells were allowed to adapt to the specified extracellular Ca^2+^ concentration by growing them first overnight, and then in a fresh culture that was allowed to reach OD_600_ = 0.4–0.5 at the start of the light exposure experiment. Msn2p nuclear localization increased at 5 mM and 10 mM compared to standard conditions (0.9 mM Ca^2+^). Nuclear localization of Msn4p increased after 30 minutes of light exposure (5 mM and 10 mM). The curves have been smoothed with an averaging filter for visualization purposes. (B) Median values of the time to first nuclear localization as a function of extracellular Ca^2+^ concentration. (G) Msn2-GFP nuclear localization trajectory classification. Cells were manually classified as either: No response (Msn2p located only in the cytoplasm), Only oscillating (Msn2p oscillates continuously, never exhibited sustained nuclear localization), or Permanent nuclear localization (Msn2p resided in the nucleus for a longer period of time towards the end of the experiment). The latter class increased drastically at 5 mM Ca^2+^. [Ca^2+^]: 0.8×10^−3^ mM (0.4 mg/l from calcium pantothenate, without CaCl_2_), 0.9 mM (standard medium), 5 mM and 10 mM.

To investigate if the Crz1p response to light exposure is influenced by extracellular Ca^2+^ concentrations, cells were grown in medium with Ca^2+^ concentrations ranging from trace amounts to moderate levels (0.8×10^−3^ mM, 0.9 mM, 5 mM and 10 mM) and then illuminated (standard synthetic defined media contains 100 mg/l CaCl_2_ (∼0.9 mM) [43]). Importantly, at these calcium concentrations no cells exhibited Crz1p nuclear localization at the start of the light exposure experiment, in contrast to the homogenous nuclear localization (i.e. most cells responded simultaneously) following a sudden exposure to high extracellular Ca^2+^ levels (50–300 mM) [2]. In that study the initial nuclear localization of Crz1p in response to Ca^2+^ was transient and it was followed by uncorrelated nuclear localization bursts in individual cells, that is, a completely different behaviour compared to the permanent localization observed here. Nevertheless, the light-induced response of Crz1p was clearly dependent on the Ca^2+^ concentration in the medium. As shown in Figs. 4A–B and Fig. S3, Crz1p localized much quicker to the nucleus with increasing Ca^2+^ concentration. The median time to first nuclear localization was shifted from 32 minutes at 0.8×10^−3^ mM Ca^2+^ to 7 minutes at 10 mM Ca^2+^ (Fig. 4B). At lower Ca^2+^ concentrations it is noticeable that the spread in time to first nuclear localization between different cells increased, i.e. the population became less homogenous (indicated by a more gentle slope, Fig. 4A). The effect of lower extracellular Ca^2+^ levels was investigated also in yeast cells grown in standard medium with the addition of 5 mM EGTA just prior to the light exposure experiment. This resulted in a drastic increase in cellular response time, even higher than that seen at 0.8×10^−3^ mM Ca^2+^ above and with even fewer cells responding at all (Fig. S4). However, in none of these cases did we observe nucleocytoplasmic oscillations appearing in response to increasing calcium levels (see also Fig. S3).

The results above indicate that extracellular uptake of Ca^2+^ is important for a fast response of Crz1p. We therefore investigated the role of the high-affinity calcium channel, Cch1p [44], [45]. Cch1p is located to the plasma membrane and is thought to be partly responsible for Ca^2+^ influx. In a *cch1Δ* strain, we found that the median time to first nuclear localization of Crz1p increased from 19 minutes in wild-type cells to 28 minutes, which suggests that Cch1p-mediated Ca^2+^ influx modulates the Crz1p response (Figs. 4C–D). We then investigated if altered extracellular Ca^2+^ concentration also affected Msn2p and Msn4p localization during light-induced stress (Figs. 4E–F). Interestingly, only a relatively small change in the overall fraction of cells with nuclear localization was observed for both transcription factors at 0.8×10^−3^ mM Ca^2+^ compared to standard conditions at 0.9 mM Ca^2+^. In addition, there was no change in the time to first nuclear localization at any of the Ca^2+^ concentrations tested (Fig. 4B). However, at 5 mM and 10 mM Ca^2+^, the overall response increased (Figs. 4E–F and Fig. S3). In the case of Msn4p the change was still quite small, but we did observe an increase in the fraction of cells with nuclear localization after 30 minutes of light exposure with no noticeable difference between 5 mM and 10 mM Ca^2+^. Msn2p exhibited a significant increase in nuclear localization, which was strongest at 5 mM Ca^2+^. This can be assigned to a higher amount of cells reaching permanent nuclear localization (Fig. 4G and Fig. S3). The higher level of transcription factors in the nucleus in response to higher calcium levels indicates that Ca^2+^ levels affects multiple signalling pathways and/or sets the thresholds for the light-induced stress responses at another level. Altogether, these results demonstrate that changes in cellular environment can affect the light-dependent response quite dramatically.

### 3.4 Internal Ca^2+^ Stores Boost Light-induced Crz1p Nuclear Localization

**Figure 5:**
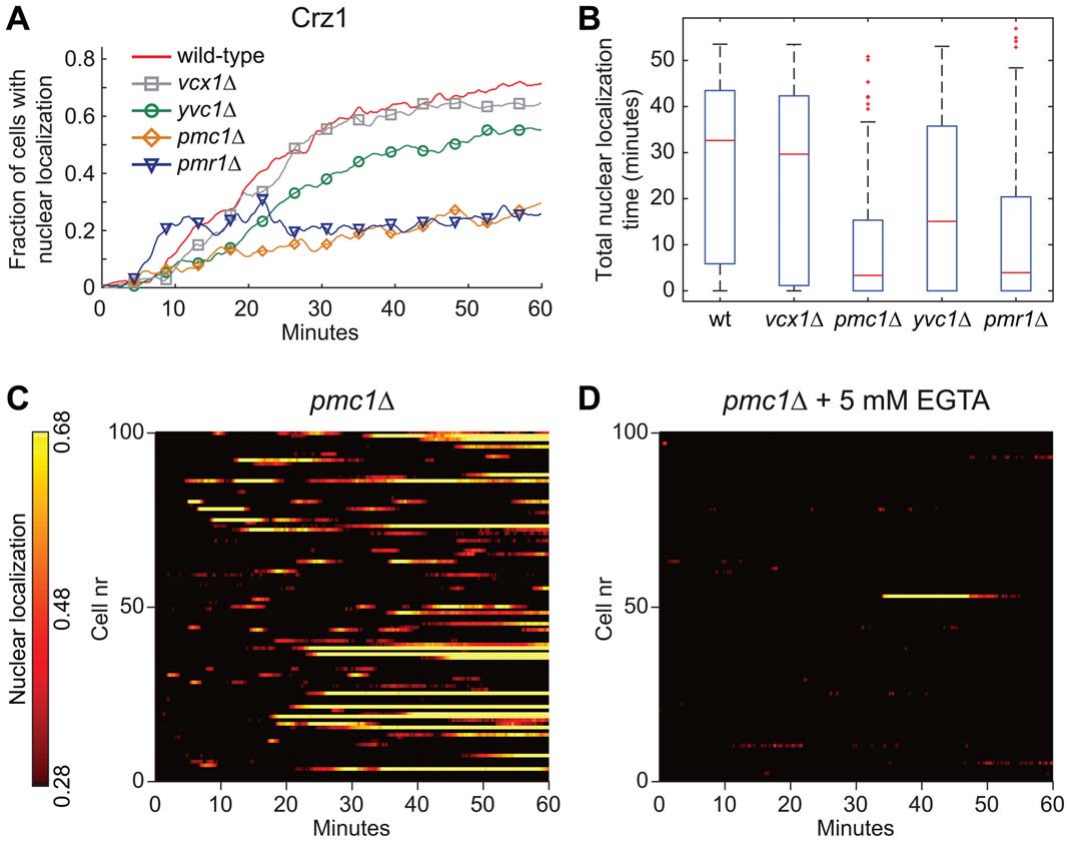
Internal Ca^2+^ pumps are important for the wild-type light-induced response of Crz1p. (A) Fraction of cells with Crz1-GFP localized to the nucleus as a function of time during continuous exposure to blue light (450–490 nm, 115 µW). (B) Total nuclear localization time of Crz1p during 60 minutes of light exposure. (C)–(D) Nucleocytoplasmic localization profiles of Crz1p extracted from fluorescence microscopy images of *pmc1Δ* cells. (D) EGTA was added to the medium to a final concentration of 5 mM just prior to exposure to blue light.

In budding yeast, the vacuole is the main reservoir for Ca^2+^, which can be released to the cytoplasm in response to certain stimuli. 90% of the total Ca^2+^ in yeast cells is located in the vacuole and most of it (roughly 99%) is bound to polyphosphate [46]. Vcx1p, Pmc1p and Yvc1p regulate the Ca^2+^ exchange between the vacuole and the cytoplasm. Pmc1p is the main vacuolar influx pump in *S. cerevisiae* and the major contributor to vacuolar Ca^2+^ levels [47], and cells lacking *PMC1* only carry ∼10% of normal vacuolar calcium levels [20]. Vcx1p is a Ca^2+^/H^+^ exchanger that have been identified to mainly transport Ca^2+^ into the vacuole [46] and to be inhibited by calcineurin [48]. Yvc1p is an ion channel that release Ca^2+^ from the vacuole [49] in response to e.g. hyperosmotic shock or hydrogen peroxide treatment [50]. To test if the vacuole was involved in controlling the response to illumination by regulating cytoplasmic Ca^2+^ levels, we studied Crz1p nucleocytoplasmic responses in strains lacking either one of *PMC1*, *VCX1* or *YVC1* (Fig. 5A). Interestingly, *pmc1Δ* cells exhibited a very different nucleocytoplasmic localization pattern compared to wild-type cells, where the essentially permanent nuclear localization behaviour of Crz1p in many mutant cells was replaced by sporadic long oscillations (Fig. 5C). The total nuclear localization time also decreased severely in the *pmc1Δ* strain (Fig. 5B). In addition, by adding 5 mM EGTA to the external medium just prior to the start of the light exposure, Crz1p did not enter the nucleus at all (Fig. 5D and Fig. S4). Taken together this indicates a combined importance of vacuolar and extracellular Ca^2+^ in the light-induced Crz1p response. Cells lacking *VCX1* displayed a pattern similar to wild-type cells, while *yvc1Δ* cells displayed a decrease in nuclear localization compared to wild-type cells (Figs. 5A–B).

The main supplier of Ca^2+^ to the endoplasmic reticulum (ER), Pmr1p, is localized to the Golgi complex [51]. In *S. cerevisiae* the ER contain up to 100-fold more Ca^2+^ than the cytosol, which can be released to the cytosol to initiate a Ca^2+^-signalling response under certain conditions [20]. Proper function of Pmr1p also seems to be important during light-induced stress, since Crz1p localized to the nucleus much quicker, but not to the same extent in *pmr1Δ* mutant cells compared to wild-type cells (Figs. 5A–B and Fig. S5).

We have previously observed altered vacuolar morphology in response to light [7]. We here extend that observation and investigated if the two signalling pathways characterized to be of importance in the light-response in this study, Ca^2+^-calcineurin-Crz1 and cAMP-PKA-Msn2/4, were involved in these light-induced vacuolar changes. During continuous illumination of the wild-type strain, the number of cells exhibiting altered vacuolar morphology, seen as a higher contrast than prior to light stress, increased with time (Figs. S6A-B). Deletion of neither *CNB1* nor *PDE2* in cells expressing Crz1-GFP altered the onset of vacuolar alterations (Figs. S6B-C). The same held true in cases where the nuclear localization of Crz1p was either increased (10 mM CaCl_2_ in the medium) or decreased by the addition of EGTA (Figs. S6E-F). Deletion of *PMC1* increased the number of cells with altered vacuolar morphology (higher contrast) already from the start of the experiment (Fig. S6D), but did not increase Crz1p localization prior to illumination (Figs. 5A and C). We conclude that altered vacuolar morphology seen upon illumination with blue light does not seem to be linked to the Ca^2+^-calcineurin-Crz1 signalling cascade or to reduced PKA activity.

### 3.5 Illumination with Blue Light Increases Levels of Cmk2 in a Manner Dependent on *CRZ1* and *de novo* Transcription

**Figure 6:**
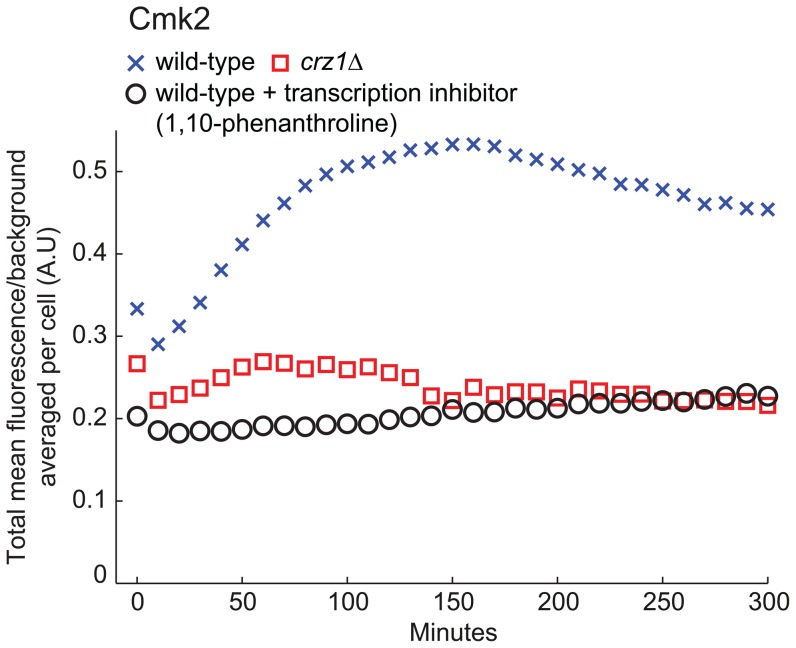
Illumination increases Cmk2-GFP fluorescence in a manner dependent on Crz1p and *de novo* transcription. Increase in Cmk2-GFP average fluorescence (population average per cell, N_cell,wt_ = 107, N_cell,*crz1Δ*_ = 99, N_cell,transcription inhibitor_ = 89) after 40 minutes of exposure to blue light (115 µW, 450–490 nm). To verify that the fluorescence increase in wild-type cells was due to *de novo* transcription of *CMK2-GFP* the transcription inhibitor 1,10-phenanthroline (100 µg/ml) was added just prior to illumination.

Finally we turned to the question whether light-induced nuclear localization of Crz1p leads to increased expression of downstream target genes or not. We used a Cmk2-GFP fusion that previously has been used as an indicator of calcineurin- and Crz1-dependent transcription [2] to address this issue. A wild-type strain and a *crz1Δ* strain expressing Cmk2-GFP were illuminated for 40 minutes (115 µW) after which the average cellular fluorescence was tracked over time (Fig. 6). The wild-type strain showed a clear increase in fluorescence, while the *crz1Δ* strain did not. In addition, by inhibiting transcription through 1,10-phenanthroline just prior to illumination, wild-type cells did not exhibit increased Cmk2-GFP fluorescence, indicating that the increase in fluorescence is dependent on *de novo* transcription. Increased fluorescence was also not seen following 180 minutes of keeping the wild-type Cmk2-GFP strain in the dark (data not shown). Taken together these data clearly show that blue light illumination triggers increased transcription of *CMK2* in a *CRZ1*-dependent manner.

## Discussion

**Figure 7:**
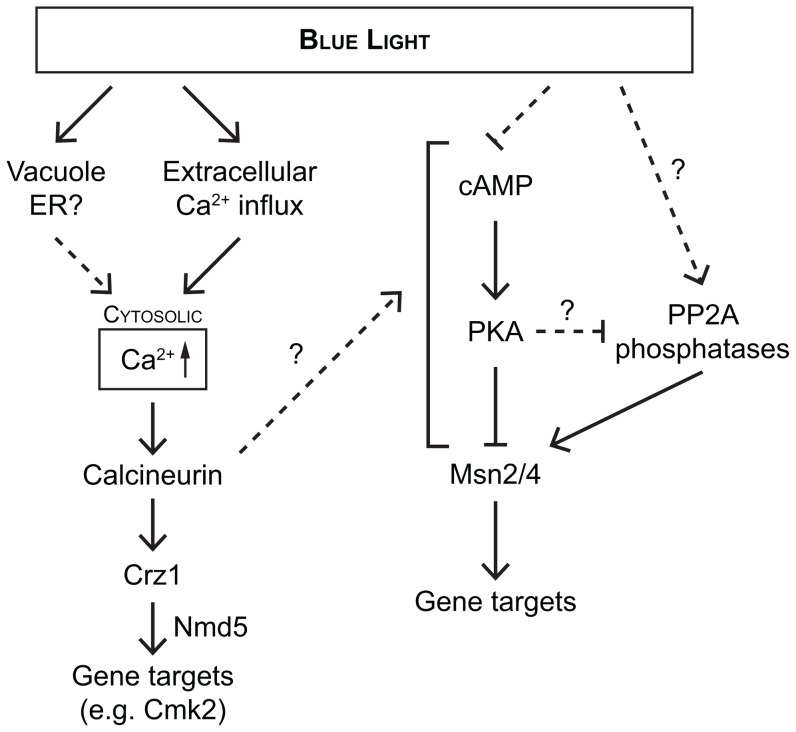
Schematic overview of the two signalling pathways responsive to illumination. This study has identified that both intra- and extracellular Ca^2+^-depots are important for the wild-type Crz1p response during illumination. Importantly, the Crz1p nuclear localization is calcineurin dependent and PKA independent.

Earlier studies have established that nuclear localization of Crz1p following stress conditions other than illumination is calcineurin-dependent [21], [35]. However, PKA also negatively regulates the nuclear localization of Crz1p [30] and previous studies show that nuclear localization of the transcription factors Msn2p and Msn4p during illumination requires reduced PKA activity. Here we find that Crz1p nuclear localization during light-induced stress is completely dependent on calcineurin (Fig. 3; *cnb1Δ* strain) and independent of the PKA activity (Fig. 3; *pde2Δ* strain). Thus, this is in stark contrast to Msn2p, which is dependent on PKA activity for light-induced nuclear localization but is unaffected in a *cnb1Δ* strain. However, we also note that light-induced Msn2p nuclear localization could possibly be regulated also by mechanisms other than the lack of inhibitory PKA phosphorylation, since it has previously been reported that deletion of the PP2A phosphatase genes *PPH21* and *PPH22* abolished Msn2p nuclear localization during light-induced stress [4], indicating that the relative activities of both phosphatases and kinases govern Msn2p nuclear localization. Recent data indicate that PKA is required for glucose-induced activation of PP2A [52], whereas mechanisms underlying the apparent PP2A requirement for Msn2p to translocate to the nucleus in response to light remain to be characterized. Furthermore, Crz1p nuclear localization during illumination was dependent on the transport factor Nmd5p, as in the case of Ca^2+^ stress. Previous studies have shown that nuclear accumulation of Crz1p in response to high extracellular Ca^2+^ requires the nuclear importin Nmd5p, but that Crz1p dephosphorylation by calcineurin also down-regulates its nuclear export [38], [53]. These findings indicate that two largely independent signalling pathways respond to continuous illumination with blue light (Fig. 7).

In addition, we demonstrate that the light-induced nuclear localization response requires relatively low levels of extracellular Ca^2+^, especially in the case of Crz1p and Msn2p. The nuclear localization profile during illumination changed differently for these two transcription factors in relation to the Ca^2+^ concentration. For Crz1p the time to nuclear localization was shortened with higher Ca^2+^ concentration (Fig. 4B), whereas Ca^2+^ did not affect the response time for Msn2p. Instead, in the case of Msn2p more cells exhibited permanent nuclear localization. At 10 mM Ca^2+^, however, Msn2p localization was somewhat reduced compared to 5 mM (Fig. 4E and Fig. S3). This could be a result of increased Msn2p degradation in the nucleus upon activation of Crz1p [54]. Degradation of Msn2p was proposed to be vital for avoiding prolonged growth arrest during constitutive stress conditions [55], as is the case here, but to play no role during short-term fluctuating conditions. However, the boost in nuclear localization of both Msn2p and Msn4p in response to blue light by increasing Ca^2+^ levels indicates that Ca^2+^ also can potentiate the Msn2p and Msn4p responses. In the case of Crz1p, we identified the plasma membrane calcium channel Cch1p to be at least partly responsible for the influx of Ca^2+^ into the cell during light-induced stress (Figs. 4C–D). Furthermore, it has been reported that calcineurin activity is higher in rich medium, which contains higher concentrations of Ca^2+^ compared to minimal medium. However, the activation of calcineurin-dependent transcription by glucose-induced calcium influx was counteracted by the availability of calcium in the medium [56]. Calcineurin controls the activity of Crz1p, which is thought to control transcription of calcineurin-dependent targets genes [12]. Hence, the quicker Crz1p response at higher extracellular Ca^2+^ concentrations could possibly be assigned to both increased basal calcineurin activity as well as increased calcium influx.

This study has implicated two signalling pathways and output transcription factors in the response to illumination, one through calcineurin and Crz1p and one through PKA and Msn2p/Msn4p. It is certainly of interest to identify the photochemical products produced upon illumination with blue light, as well as components responsible for sensing an enhanced photon-induced toxicity upstream of the signalling pathways. The rapid sustained nuclear localization of Crz1p could indicate that the stress caused by illumination is severe and more difficult to adapt to than the level of stress caused by 200 mM extracellular Ca^2+^ [2], which results in stochastic “bursts” of Crz1p nuclear localization and for which cells are equipped with adequate regulatory systems. One possible source of light-induced toxicity that has been suggested is the production of H_2_O_2_ via flavin-containing oxidases [57]. Binding elements for Crz1p [12], as well as Msn2p and Msn4p [11], have been found in promoters of target genes that are induced following exposure to H_2_O_2_. This could possibly, to some extent, explain the reason for the observed light-induced nuclear localization of Crz1p, Msn2p, and Msn4p. Addition of sublethal doses of H_2_O_2_ to the culture medium has been shown to induce influx of Ca^2+^ to the cytosol from both extracellular and vacuolar sources, whereas lethal doses primarily induce efflux from the vacuole [50]. Under these conditions Yvc1p was required for vacuolar Ca^2+^ release. Our data indicate that both extracellular and intracellular Ca^2+^ depots are mobilized during exposure to blue light and we previously suggested that the amount of toxic photoproducts increases with time during continuous illumination [3]. The early Crz1p response seems to be dependent primarily on extracellular Ca^2+^-availability, since cells lacking the plasma membrane calcium channel Cch1p displayed a delayed response, i.e. nuclear localization of Crz1p. In addition, decreased extracellular Ca^2+^ concentrations or the addition of EGTA also increased the response time. The quick response at 10 mM Ca^2+^, which might indicate increased extracellular influx, stands in contrast to decreased extracellular influx of Ca^2+^ observed following amiodarone treatment of cells grown at the same Ca^2+^ level [58]. This influx was, however, not mediated by Cch1p and might therefore involve other unknown Ca^2+^ entry pathways. The level of stress increases with illumination time (increased accumulation of phototoxic products) and therefore the combined effect of intracellular and extracellular Ca^2+^ stores might become increasingly important. Our results show that intracellular stores in the vacuole also affected the ability of cells to respond to illumination. In *pmc1Δ* cells, carrying only ∼10% of normal vacuolar Ca^2+^ amounts [20], the Crz1p response was much weaker than in wild-type cells and furthermore this response in *pmc1Δ* cells was lost completely by adding EGTA. The decreased Crz1p nuclear localization in cells lacking the vacuolar Ca^2+^-efflux pump Yvc1p (Fig. 5) also, at least to some extent, supports this hypothesis. Furthermore, previous studies showed that *pmr1Δ* cells display perturbed Ca^2+^ homeostasis by having a higher Ca^2+^ influx, increased cytosolic Ca^2+^ concentration and indeed higher calcineurin activity [59]. The quicker response in *pmr1Δ* cells, i.e. the decreased time it takes for Crz1-GFP to enter the nucleus in this study is consistent with an increased basal calcineurin activity, higher influx rate and already elevated cytosolic calcium levels. The lower amount of nuclear localization and cells responding might result from altered subcellular Ca^2+^ distribution, since e.g. Pmc1p was reported strongly upregulated and mislocalized in the *pmr1Δ* mutant [60], possibly a response to compensate for secretory defects associated with the lack of Pmr1p [47]. However, due to the complexity of the response there is room for further investigations in order to get a broader understanding of Ca^2+^-dependent mechanisms in the light-phenomenon.

The roles of Msn2p and Msn4p in stress responses have recently been shown to be both gene and condition specific, and to have a major role in acquired stress tolerance [1]. It was also hypothesized that Msn2p and Msn4p are activated differently in response to different stresses; deletion of *MSN2* decreases gene expression of various target genes, while deletion of *MSN4* does not. However, a double deletion results in even lower gene expression, indicating some level of redundancy and that Msn4p contribute to transcriptional induction of certain genes in concert with Msn2p [14], [28], [61]. Our data clearly show that Msn2p and Msn4p display different responses to the same type and level of light-induced stress. In addition, based on the number of cells with nuclear localization, Msn4p nuclear localization cannot be present in all cells that exhibit Msn2p nuclear localization during 60 minutes of light exposure. Both transcription factors responded within the same time (Fig. 2), but Msn4p seems to accumulate in the nucleus mainly during the early stages of the stress response (Figs. 1 and 2). This data further substantiate the different roles of Msn2p and Msn4p and are also inline with a previous publication that demonstrated different levels of hyperphosphorylation in these two proteins upon heat stress and nutrient depletion [27]. One could hypothesise that Msn4p is more important at the early phase of stress adaptation, at even higher stress levels or in situations were combinations of multiple stresses are present. The slight increase in nuclear localization of Msn4p upon combined effects of increased calcium concentration and light stress (Fig. 4F) could possibly point towards the latter.

In this work we have quantified the nuclear localization dynamics of the transcription factors Crz1p, Msn2p and Msn4p as a function of light-induced cellular stress and investigated and discussed the connection to Ca^2+^ signalling in yeast. The three proteins exhibit distinctly different stress responses. In particular, Crz1p displays a rapid and permanent nuclear localization that leads to transcription of its target gene *CMK2* in a *CRZ1*-dependent manner. Increased extracellular Ca^2+^ levels leads to a stronger light-induced response for all three transcription factors. Studies in mutants lacking Ca^2+^ transporters indicate that influx of extracellular Ca^2+^ is crucial for the initial stages of light-induced Crz1p nuclear localization, while mobilization of intracellular Ca^2+^ stores appears necessary for a sustained response. We also found that Crz1p nuclear localization is dependent on calcineurin and the carrier protein Nmd5p, but independent on PKA activity. The nuclear localization patterns of Msn2p and Msn4p, which are characterized by stochastic oscillations, are instead strongly dependent on PKA activity. We conclude that two central signalling pathways, cAMP-PKA-Msn2/4 and Ca^2+^-calcineurin-Crz1, are both activated by blue light illumination. However, the proximal causes and detailed mechanisms behind light-induced stress in yeast is still an open issue.

## Supporting Information

Figure S1 **Msn2p nuclear localization in** ***cnb1*** **Δ cells.** The total time Msn2p spent in the nucleus during 60 minutes of continuous light exposure (115 µW, 450–490 nm) in a *cnb1Δ* strain. *CNB1* encodes the regulatory subunit of calcineurin. The two populations are not significantly different (p-value = 0.08, Mann-Whitney U-test), but the *cnb1Δ* strain displays a tendency towards decreased Msn2p nuclear localization.
(EPS)

Figure S2 **Increased PKA activity inhibits Msn2p and Msn4p nuclear localization during illumination.** Nucleocytoplasmic localization profiles of (A) Msn2p and (B) Msn4p in a *pde2Δ* strain (high PKA activity). Cells were illuminated with continuous blue light (115 µW, 450–490 nm) during 60 minutes.
(TIF)

Figure S3 **The effect of Ca^2+^ concentration on the nucleocytoplasmic localization responses.** Nucleocytoplasmic localization profiles extracted from fluorescence microscopy images for Crz1-GFP (left column), Msn2-GFP (middle column) and Msn4-GFP (right column) at four different Ca^2+^ concentrations (rows). [Ca^2+^]: 0.8×10^−3^ mM, 0.9 mM (standard medium), 5 mM and 10 mM. Cells were exposed to continuous blue light illumination (450–490 nm, 115 µW) during 60 minutes and the nuclear localization of the transcription factors were analysed. No cells displayed nuclear localization at the start of the experiment as a result of the altered Ca^2+^ concentration compared to standard medium. Note that Ca^2+^ can potentiate the response in particular for Crz1p and Msn2p.
(TIF)

Figure S4 **Ca^2+^ chelation by EGTA inhibits the nuclear localization response of Crz1p during illumination.** Addition of EGTA to the culture medium to a final concentration of 5 mM just prior to illumination increases the response time and lowers the number of cells with nuclear localization of Crz1p (blue curve) compared to wild-type cells (red curve) or cells grown in trace amounts of Ca^2+^ (black curve). In a *pmc1Δ* strain, Crz1p did not localize to the nucleus (green curve), indicating that both extracellular Ca^2+^-influx and vacuolar stores are important for the wild-type response.
(EPS)

Figure S5 **Crz1p nuclear localization in** ***pmr1Δ*** **cells.** (A) The nucleocytoplasmic localization profile of Crz1-GFP extracted from fluorescence microscopy images. Cells were continuously illuminated with blue light (450–490 nm, 115 µW). (B) Time to first Crz1p nuclear localization. Deletion of *PMR1* decreases the response time of Crz1p. The differences are statistically significant (p<0.001; Mann-Whitney U-test).
(TIF)

Figure S6 **Altered vacuolar morphology seems independent of perturbations in the Ca^2+^-calcineurin-Crz1 signalling cascade or in PKA activity.** (A) Altered vacuolar morphology in response to illumination (450–490 nm, 115 µW) was quantified by manual inspection in (B) wild-type and *cnb1Δ* cells, (C) *pde2Δ* cells, (D) *pmc1Δ* cells, (E) cells treated with 5 mM EGTA just prior to light-induced stress and (F) cells grown in higher extracellular Ca^2+^ levels. The plots show the mean value of two independent experiments with error bars representing 1 standard deviation. These data indicate that altered vacuolar morphology seen upon illumination with blue light does not seem to be linked to the Ca^2+^-calcineurin-Crz1 signalling cascade or to reduced PKA activity.
(EPS)

Datasheet S1 **Experimental data.** Nuclear localization trajectories for individual cells.
(XLSX)

Datasheet S2 **Experimental data.** Nuclear localization trajectories for individual cells.
(XLSX)

Datasheet S3 **Experimental data.** Nuclear localization trajectories for individual cells.
(XLSX)
