# Supplementary figures and images for: The Yeast Transcription Factor Crz1 Is Activated by Light in a Ca2+/Calcineurin-Dependent and PKA-Independent Manner
Source: PLoS One. 2013 Jan 15;8(1):e53404. doi: 10.1371/journal.pone.0053404 (PMC3546054; doi:10.1371/journal.pone.0053404)

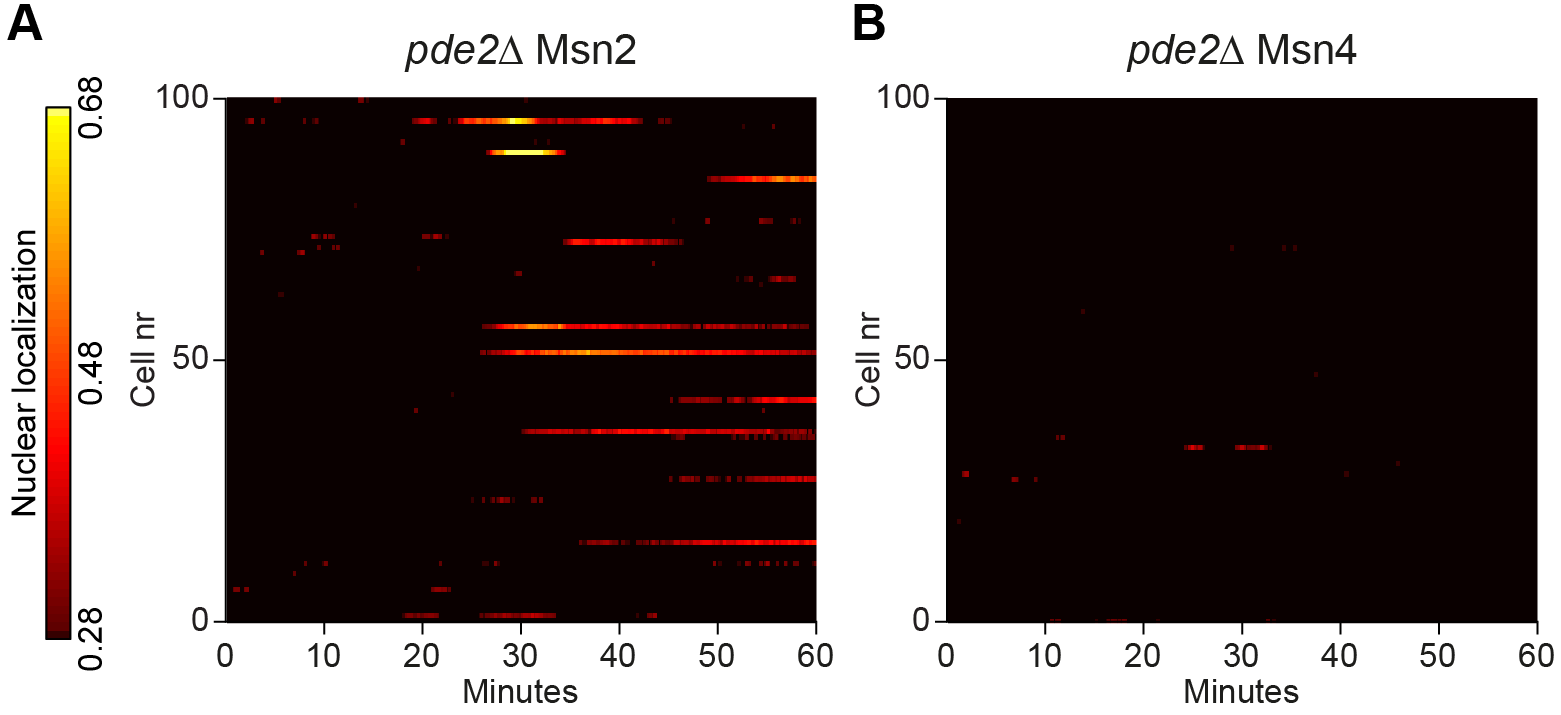

Supplement: Figure S2 — Increased PKA activity inhibits Msn2p and Msn4p nuclear localization during illumination. Nucleocytoplasmic localization profiles of (A) Msn2p and (B) Msn4p in a pde2Δ strain (high PKA activity). Cells were illuminated with continuous blue light (115 µW, 450–490 nm) during 60 minutes. (TIF) [file pone.0053404.s002.tif]

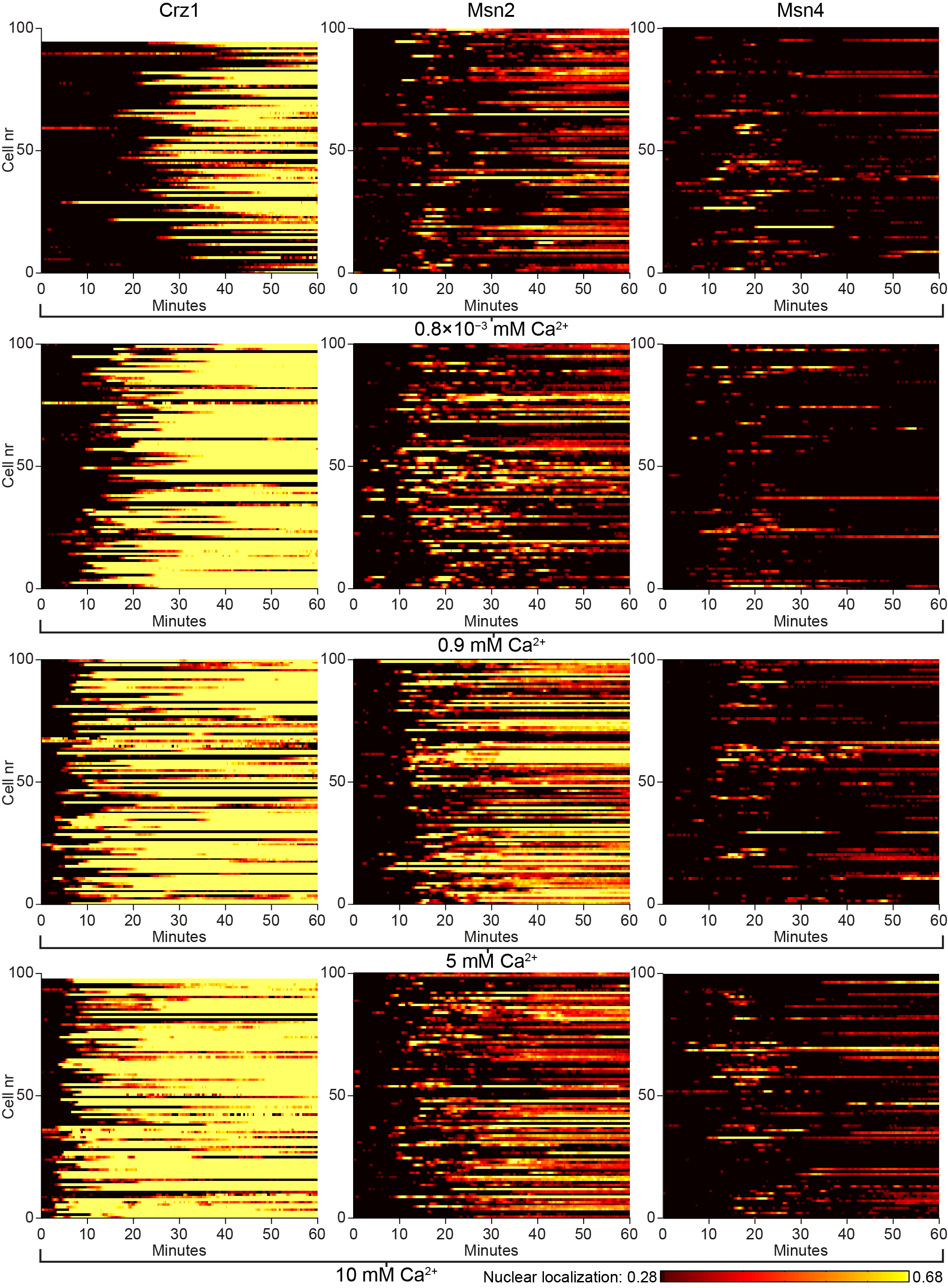

Supplement: Figure S3 — The effect of Ca2+ concentration on the nucleocytoplasmic localization responses. Nucleocytoplasmic localization profiles extracted from fluorescence microscopy images for Crz1-GFP (left column), Msn2-GFP (middle column) and Msn4-GFP (right column) at four different Ca2+ concentrations (rows). [Ca2+]: 0.8×10^−3^ mM, 0.9 mM (standard medium), 5 mM and 10 mM. Cells were exposed to continuous blue light illumination (450–490 nm, 115 µW) during 60 minutes and the nuclear localization of the transcription factors were analysed. No cells displayed nuclear localization at the start of the experiment as a result of the altered Ca2+ concentration compared to standard medium. Note that Ca2+ can potentiate the response in particular for Crz1p and Msn2p. (TIF) [file pone.0053404.s003.tif]

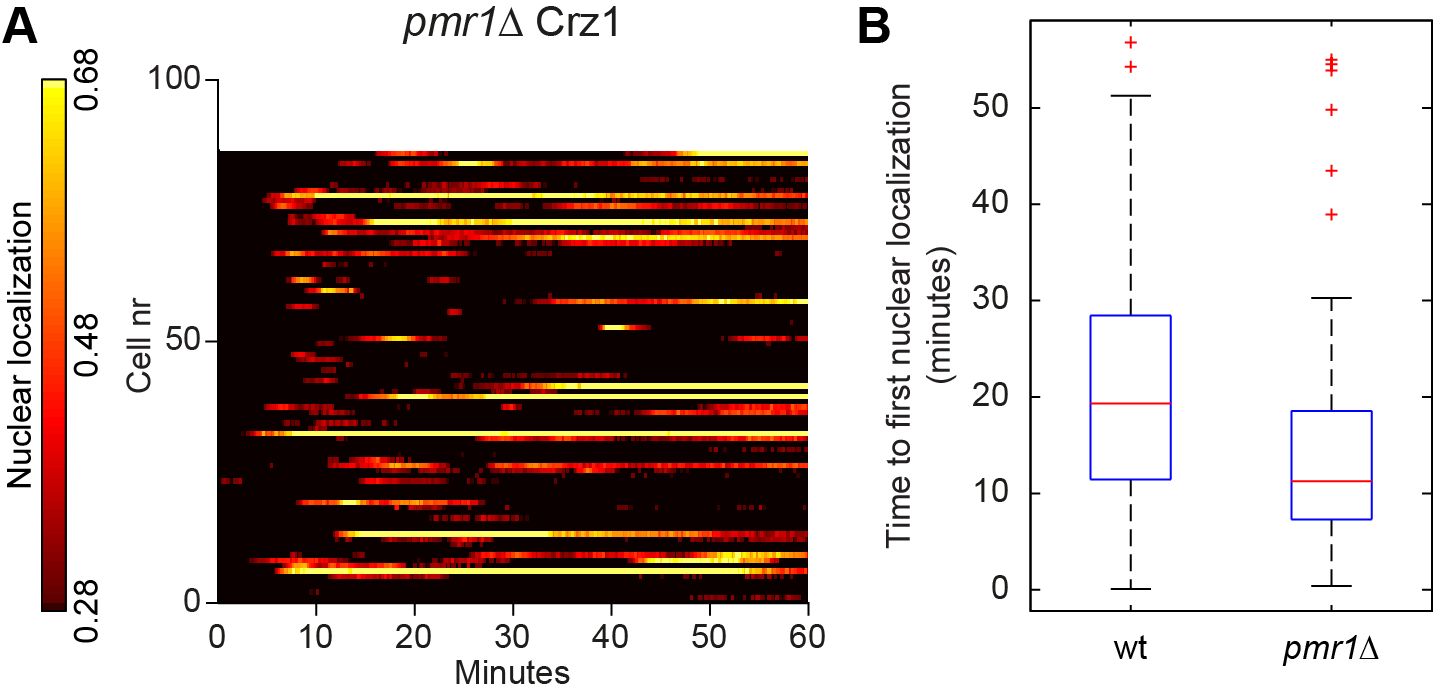

Supplement: Figure S5 — Crz1p nuclear localization in pmr1Δ cells. (A) The nucleocytoplasmic localization profile of Crz1-GFP extracted from fluorescence microscopy images. Cells were continuously illuminated with blue light (450–490 nm, 115 µW). (B) Time to first Crz1p nuclear localization. Deletion of PMR1 decreases the response time of Crz1p. The differences are statistically significant (p<0.001; Mann-Whitney U-test). (TIF) [file pone.0053404.s005.tif]
